# Identifying foraging habitats of Baltic ringed seals using movement data

**DOI:** 10.1186/s40462-015-0058-1

**Published:** 2015-09-23

**Authors:** Sari M. Oksanen, Marja Niemi, Markus P. Ahola, Mervi Kunnasranta

**Affiliations:** Department of Biology, University of Eastern Finland, PO Box 111, FI-80101 Joensuu, Finland; The Natural Resources Institute Finland, Itäinen Pitkäkatu 3, FI-20520 Turku, Finland

**Keywords:** Baltic Sea, First passage time, GPS phone tag, Habitat use, Home range, *Pusa hispida botnica*, Seal-fishery interaction

## Abstract

**Background:**

Identification of key foraging habitats of aquatic top predators is essential for designing effective management and conservation strategies. The Baltic ringed seal (*Phoca hispida botnica*) interacts with anthropogenic activities and knowledge of its spatial ecology is needed for planning population management and mitigating interactions with coastal fisheries. We investigated habitat use and foraging habitats of ringed seals (*n =* 26) with satellite telemetry in the northern Baltic Sea during autumn, which is important time for foraging for ringed seals. We used first passage time (FPT) approach to identify the areas of high residency corresponding to foraging areas.

**Results:**

Tracked seals showed considerable movement; mean (±SD) home ranges (95 % adaptive local nearest-neighbour convex hull, a-LoCoH) were 8030 ± 4796 km^2^. Two seals moved randomly and foraging areas could not be identified for them. The majority (24/26) of the studied seals occupied 1–6 main foraging areas, where they spent 47 ± 22 % of their total time. Typically the foraging areas of individuals had a mean distance of 254 ± 194 km. Most of the seals (*n =* 17) were “long-range foragers” which occupied several spatially remote foraging areas (mean distance 328 ± 180 km) or, in the case of two individuals, did not concentrate foraging to any particular area. The other seals (*n =* 9) were “local foragers” having only one foraging area or the mean distance between several areas was shorter (67 ± 26 km). Foraging areas of all seals were characterised by shallow bathymetry (median ± SD: 13 ± 49 m) and proximity to the mainland (10 ± 14 km), partly overlapping with protected areas and coastal fisheries.

**Conclusions:**

Our results indicate that in general the ringed seals range over large areas and concentrate feeding to different—often remote—areas during the open water season. Therefore, removal of individuals near the fishing gear may not be a locally effective method to mitigate seal depredation. Overlap of foraging areas with protected areas indicate that management of key foraging and resting habitats could to some extent be implemented within the existing network of marine protected areas.

**Electronic supplementary material:**

The online version of this article (doi:10.1186/s40462-015-0058-1) contains supplementary material, which is available to authorized users.

## Background

Identifying areas that are important in fulfilling different life history priorities, such as breeding and foraging habitats, is often an initial step in understanding habitat use of mobile aquatic predators, and thereby in designing effective management and conservation strategies [1, 2]. Many seal species interact with fisheries while feeding [3–5], therefore studying foraging habitats may help to assess actions to mitigate seal − fishery interactions [6, 7]. For example, marine protected areas (MPA) targeting to conserve the important feeding grounds of mobile predators have successfully mitigated negative interactions, such as by-catch and resource competition [8, 9]. Also the negative effects that pinnipeds can have on fisheries, such as damaging catches and fishing gear, could be reduced with locally focused removal when seals show strong foraging site fidelity [3, 10].

Although Arctic ringed seal (*Phoca hispida*) in general inhabits remote locations and interacts relatively little with humans, the Baltic subspecies (*P. h. botnica*) inhabits areas where human activities range over their entire distribution [11]. Hunting and reproductive problems due to environmental pollution caused the population to collapse from ~ 200 000 to only about 5000 individuals during the 20th century [12, 13]. Due to the protection of the seals and decrease in organochlorine concentrations [12, 14], the population has now recovered to circa 13 000 seals [15], and the most recent estimates indicate even larger population (census size 17 600 seals, T. Härkönen, personal communication). Ringed seals, as many other phocid seals, have three key elements during their annual cycle, i.e. breeding, moulting and foraging [16]. Ringed seals give birth, rear pups and mate during the ice-covered time and exhibit site fidelity to breeding sites [16–19]. Moulting takes place later in spring and is characterized by extended haul-out periods [20–22]. Although ringed seals do not fast during breeding or moulting, foraging is limited during breeding and extensive haul out [16, 23]. Open water season after the moult, on the other hand, is an important foraging period, and seals gain weight for the next winter [23–25]. While the Arctic ringed seal is considered quite nomadic during the open water season [16, 26–28], its land locked subspecies inhabiting Lake Saimaa (*P. h. saimensis*) is relatively sedentary throughout the year [29, 30]. Also the Baltic ringed seal are suggested to be sedentary [25], but detailed studies on its spatial ecology are lacking.

**Fig. 1:**
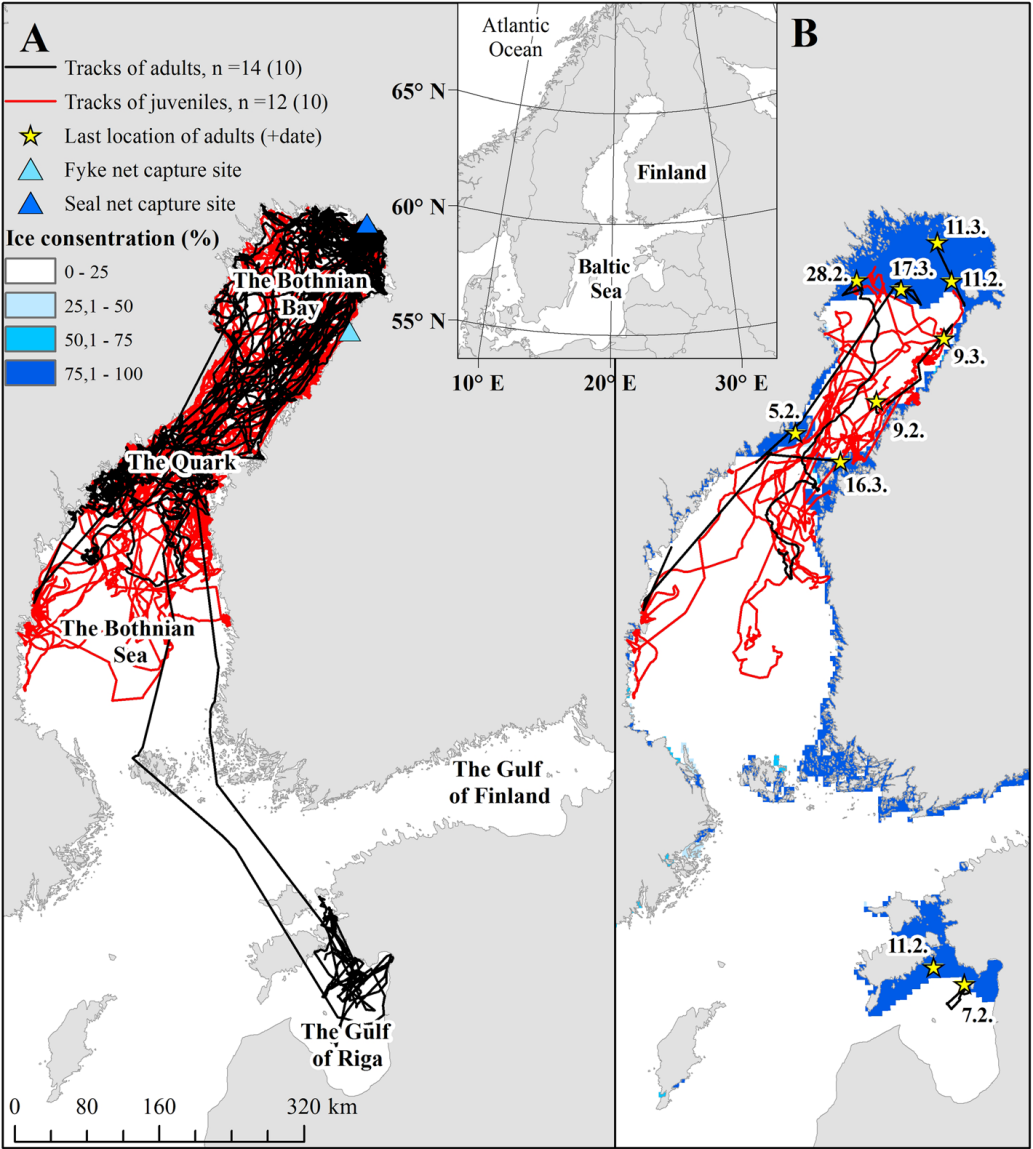
Movements of Baltic ringed seals during the whole tracking period (**a**) and during breeding time (**b**). The whole tracking period: August-May in years 2011–2014. Breeding time: February-March (number of tracked seals during breeding time is in the brackets). Mean ice concentration is for period 17.2.-2.3.2014 (data source: [71])

Approximately 75 % of the current Baltic ringed seal population inhabits the northernmost part of the Baltic Sea—the Bothnian Bay [15]. Other subpopulations in the southern breeding areas in the Gulf of Riga and Gulf of Finland (Fig. 1) are suggested to suffer from lack of suitable ice cover for breeding, and the relative importance of the Bothnian Bay as the main distribution area is expected to increase due to climate change [15, 31, 32]. The growing numbers of ringed seals in the Bothnian Bay reportedly cause substantial catch losses to coastal fisheries and means to mitigate depredation, such as removal of seals near the fishing gear, have been proposed [33–35]. Detailed knowledge of the spatial ecology of ringed seals inhabiting the Bothnian Bay is therefore needed for planning strategies for conservation and mitigation of seal-fishery conflict. Predators concentrate foraging effort in areas with the highest probability of capturing prey [36]. Therefore, identifying high residency areas of seals allow identification of key foraging habitats and thereby estimating the degree of spatial overlap between seals and coastal fisheries. In this study, we examined the habitat use of the Baltic ringed seal in the Bothnian Bay with a special focus on identifying important foraging habitats.

## Methods

### Study area

The Baltic Sea (surface area 400 000 km^2^) is a semi-enclosed brackish water system consisting of several basins (Fig. 1) and characterised by shallow bathymetry (mean depth 54 m and maximum depth 459 m) [37]. The study was mainly conducted in the Gulf of Bothnia (surface area 115 500 km^2^), which comprises the Bothnian Bay, the Quark and the Bothnian Sea (Fig. 1). The mean depth of the Gulf of Bothnia is 55 m and maximum 293 m [37].

### Animal handling and data collection

Ringed seals were captured during autumn in 2011–2013 from important coastal fishing areas in the Bothnian Bay (Fig. 1). Fyke nets (*n =* 4) were equipped with “seal socks” allowing the seals to access the surface to breathe [38] and were set for fishing by commercial fishermen from May to October-November. In addition, floating seal nets (mesh size 180 mm, height 4 m, length 80 m, net material 0.7 monofil, Hvalpsund net A/S) were used for capturing seals during October and November. The seal nets were usually anchored from both ends in areas with water depth of 5–8 m.

Seals were manually restrained, while GPS phone tags (Sea Mammal Research Unit, University of St Andrews, UK) were attached to the dorsal fur above the scapulas with two-component epoxy glue (Loctite Power Epoxy, 5 min). Only seals weighing ≥ 40 kg received tags. To ensure later identification, a uniquely numbered plastic ID-tag (Jumbo tag, Dalton, UK) was attached to the hind flipper. Sex, weight, girth, and length were recorded and individuals were divided into two age classes (juveniles and adults) according to the weight on the basis of age-weight database (Natural Resources Institute Finland). Seals with body weight over 50 kg were classified as adults (estimated age ≥ 4 years). Capturing and tagging protocol was approved by the Finnish Wildlife Agency (permit no. 2011/00082 and 2013/00197) and the Animal Experiment Board of Finland (no. ESAVI/1114/04.10.03/2011). All efforts were made to minimize the handling times and thereby the stress of the study animals.

The phone tags were programmed to attempt GPS location 2 to 3 times per hour. Tags separated between at-sea locations and haul out locations and a haul-out event began when the tag was continuously dry for 10 min and ended when wet for 40 s. The location data of the seals (*n =* 26) were filtered following McConnell et al. [39] and as a result, on average (± SD) 2.0 ± 2.9 % of individual’s locations were removed. Data of individual KU13 contained 4 outlier locations even after filtering and they were removed. To complement the GPS data, additional Argos flipper tags (SPOT5, Wildlife Computers Inc.) were deployed to four seals. Flipper tags were duty cycled to transmit 2 h during daytime and 2 h during night in 2 to 8 days per month.

### Home range analysis

Home ranges were investigated with minimum convex polygon (MCP) [40] and adaptive local nearest neighbour convex hull (a-LoCoH) analyses [41]. Home ranges (95 % of the locations in MCP and 95 % isopleths of the utilisation distribution in the LoCoH) were estimated for seals with a tracking period of over 20 days (Additional file 1: Table S1). In a-LoCoH, parameter *a* was set by taking the maximum distance between any 2 locations in each individuals’ data set [41]. For an individual MI12 utilisation distribution could not be constructed with a-LoCoH with that *a*-parameter and set of locations. As the a-LoCoH estimator is not very sensitive for changes in *a* [41], we changed it to the nearest value allowing us to estimate the utilisation distribution (from 178 144 to 178 010). Land areas were subtracted from the MCP home range estimates. Effect of age and sex on the a-LoCoH home range size was tested with univariate general linear model (size = intercept + sex + age) in SPSS Statistics 19 (IBM). Two-way interaction terms were insignificant (*p <* 0.05) and therefore excluded. Variances of model residuals were not equal between the age classes and log-transformation was therefore used.

### First passage time analyses

We investigated important foraging habitats of tracked seals between August and January. This largely coincides with the period (Jun – Dec), when Baltic ringed seals forage and gain weight more intensively than at other times of the year [25]. We hereafter refer to this mostly open water period as foraging season, with the recognition that ringed seals also forage throughout the year [42, 43]. The foraging habitats were detected with the first passage time (FPT) analyses [36]. FPT, defined as the time required for a tracked individual to cross a circle of a given radius, is a measure of animals’ search efforts along the track [36, 44]. FPT can also be used to detect any movement patterns leading to increased residency [45].

**Fig. 2:**
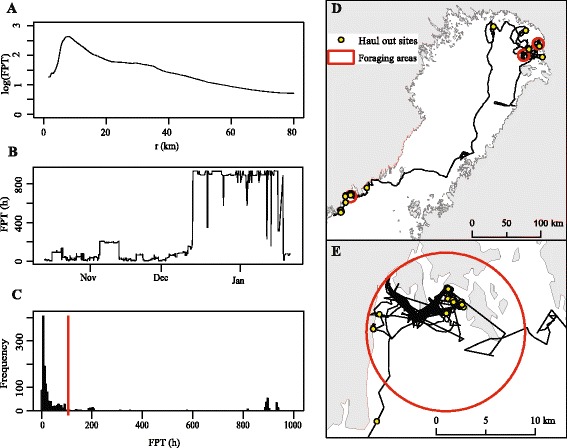
Examples of FPT analyses and foraging areas of individual AA13. **a**: variance in first passage time (FPT) as a function of radius (r). **b**: Change of FPT in time. **c**: Classification of high residency locations on the basis of the histogram (red line indicates the division). **d**: Movements, foraging areas and haul out sites. **e**: Closer look to the foraging area with the highest FPT values

The analyses were done using the AdehabitatLT package [46] in R 2.15.3 [47]. Haul out locations were included in the FPT analyses. Before the analyses, we removed possible gaps in the location data of each individual by dividing the data into several tracks when time between two consecutive locations was > 1 d. As the quality of FPT analyses depends on tracking duration [48], we dropped shortest tracking records (<15 locations, mean duration ± SD: 8.8 ± 12.3 h) from the analyses. We received on average 17 ± 8 daily locations and to ensure that points along tracks were equally represented [36], we generated locations in 1.2 km intervals (corresponding to the mean distance between consecutive GPS locations) along the tracks, assuming that animals travelled linearly and with constant speed between obtained GPS-locations. FPT values were calculated for every location with radii of the circle changing from 1.5 to 80 km (in 0.5 km increments). The optimal radius for each track were then estimated by plotting the variances of the log-transformed FPTs as a function of radius. The peak in the variance (var-max) indicates a scale at which an individual increased its search efforts [36] and the FPTs corresponding to this radius were selected (see Fig. 2a and b for an example).

### Defining foraging areas and haul out sites

To separate locations with high FPT values (high residency locations) from low, a threshold value was obtained from a histogram of FPT values for each track [49]. FPTs had multimodal distribution, where low FPTs formed one mode of the histogram and high FPTs one or several modes (see Fig. 2c for an example). The high residency locations were then used to detect one or several foraging areas within each track following the method in Lefebvre et al. [45]; first foraging area was constructed by assigning the highest FPT value as a centre of the circle with radius corresponding to var-max. Other areas were formed when the next highest FPTs with the associated circle did not overlap with another foraging area. According to the number and locations of these areas, the seals were then classified to “local foragers” and “long-range foragers”. Local foragers had only one foraging area or the maximum distance between centroids of different areas was ≤ 121 km (corresponding to the two adjacent foraging areas with the largest observed var-max of 60.5 km). Long-range foragers either occupied several separate foraging areas with a maximum distance of >121 km or did not show increasing search effort (no var-max detected) and, therefore, foraging areas could not be identified.

Haul out sites were defined from the GPS locations. Location error and small scale changes in the haul out place were taken into account by defining all locations that were within 50 m of each other as one haul out site. Time budget and diurnal rhythm of haul out were constructed on the basis of summary data provided by GPS phone tag, which reports percent of haul out, diving and being near the surface (threshold 1.5 m) in two hours bouts.

### Foraging habitat characteristics

To investigate the characteristics of foraging habitat, the depth and distance to the coastline of high residency locations were calculated using bathymetric raster data (grid size 250 × 250 m) and catchment area data [50]. To examine the overlap of the foraging habitats with protected areas, we calculated the percentage of high residency locations of the seals within the MPAs designated by the Helsinki Comission (HELCOM [50]) and Natura 2000 sites [51] that are protected under the European Union’s Habitats Directive [52]. Overlapping MPAs and Natura 2000 sites can be of different shape and size depending on the targets of protection, as the Natura 2000 network protects habitats and species at EU level and the HELCOM MPAs network at the level of the Baltic Sea. To get an overview of the overlap of seals and important coastal fishing areas, we used a dataset of annual catches (in tons of kg) of commercial coastal fisheries in year 2007 [50]. We calculated the percentage of high residency locations within 50 × 50 km grids (corresponding to ICES statistical rectangles) in which the annual catch were above the median value for the Baltic Sea.

## Results

### Telemetry performance and home range size

In total, 26 out of the 61 live-captured ringed seals were heavy enough (≥40 kg) to be equipped with GPS phone tags. Tagged seals captured with fyke nets (in Aug-Nov) were mainly young (9/10 individuals) whereas seals captured with nets (in Oct-Nov) were mostly adults (13/16, Additional file 1: Table S1). Juveniles were on average (±SD) tracked for longer periods than adults (156 ± 31 days and 86 ± 33 days, respectively; Table 1). Two tags (for adults EL11 and PI12) only functioned < 20 days and these data sets were therefore excluded from the home range analyses. The average number of GPS locations per tracking day was 17 ± 8. Three out of four flipper tags functioned and provided data (21–97 total locations) from tagging until May extending the overall tracking period by two to three months (Additional file 1: Table S1).

**Table 1:** Summary of the tag performance of the Baltic ringed seals equipped with GPS phone tags. Dur = duration of tracking period (d). Locs = number of obtained GPS locations

			Whole tracking period			Foraging season (Aug-Jan)		Breeding season (Feb-Mar)	
		Weight (kg)	dur	locs	locs/d	dur	locs	dur	locs
Juveniles	Mean	43	156	2524	16	112	1959	43	608
	SD	3	31	1571	8	26	1293	22	521
	*n*		*12*			*12*		*10*	
Adults	Mean	91	86	1346	17	68	1305	14	57
	SD	19	33	771	9	22	686	16	161
	*n*		*14*			*14*		*10*	

During the whole tracking period (August-May), tracked seals ranged over large areas in the Bothnian Bay and the Bothnian Sea (Fig. 1a); mean maximum distance from capture sites being 392 ± 195 km (measured as great-circle distance between the capture site and the utmost location point). Mean a-LoCoH home range size for juveniles was 8721 ± 6177 km^2^ and for adults 7339 ± 2983 km^2^ (Table 2). Juveniles had considerably greater individual variation among their home range sizes than adults (Levene’s test, F = 7.742, *p =* 0.011). However, we did not detect any age or sex dependent differences on the a-LoCoH home range sizes (for age *p =* 0.900 and for sex *p =* 0.513, R^2^ = 0.021). Two adult females (HE11 and II11) migrated to the Gulf of Riga (maximum distance from capture site 888 and 798 km, respectively) in late November—early December and were located there until the end of tracking in February.

**Table 2:** Estimated home range sizes (km^2^) of the Baltic ringed seals

		Home range (MCP 95 %)			Home range (a-LoCoH 95 %)		
	N	Mean	SD	Range	Mean	SD	Range
Juveniles	12	31664	18777	5289–66937	8721	6177	727–18899
Adults	12	31466	15045	12852–61882	7339	2983	1132–12280
Males	9	28601	18415	5289–66937	7297	5220	727–18899
Females	15	33343	15878	6431–61882	8470	4654	1132–17565
Total	24	31565	16640	5289–66937	8030	4796	727–18899

Tracking of many adults ended likely when they moved to the ice-covered areas, and the locations data of adults are therefore scarce during the breeding season in February-March (Table 1). The last obtained locations from GPS phone tags and additional locations from flipper tags indicate that adults were mostly located in the ice-covered areas in the Bothnian Bay and two also in the Gulf of Riga (Fig. 1b), which are also important breeding areas. The juveniles were moving mostly in open-water areas and near the ice-edge (Fig. 1b).

### Foraging areas and haul out sites

**Fig. 3:**
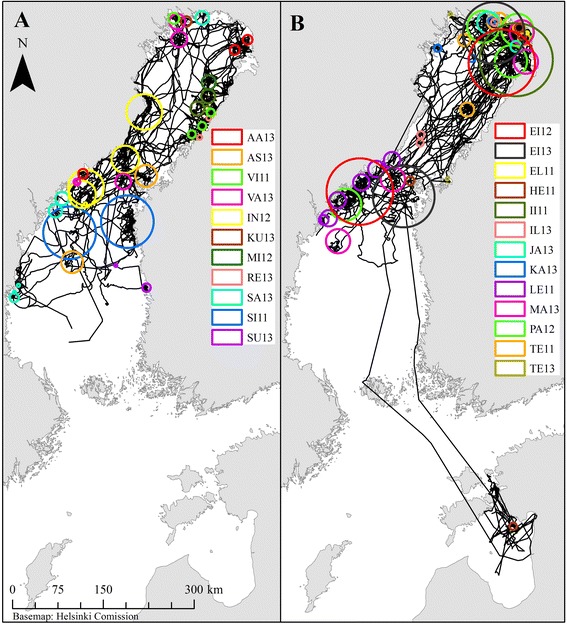
Foraging areas for juvenile (**a**) and adult (**b**) Baltic ringed seals

**Fig. 4:**
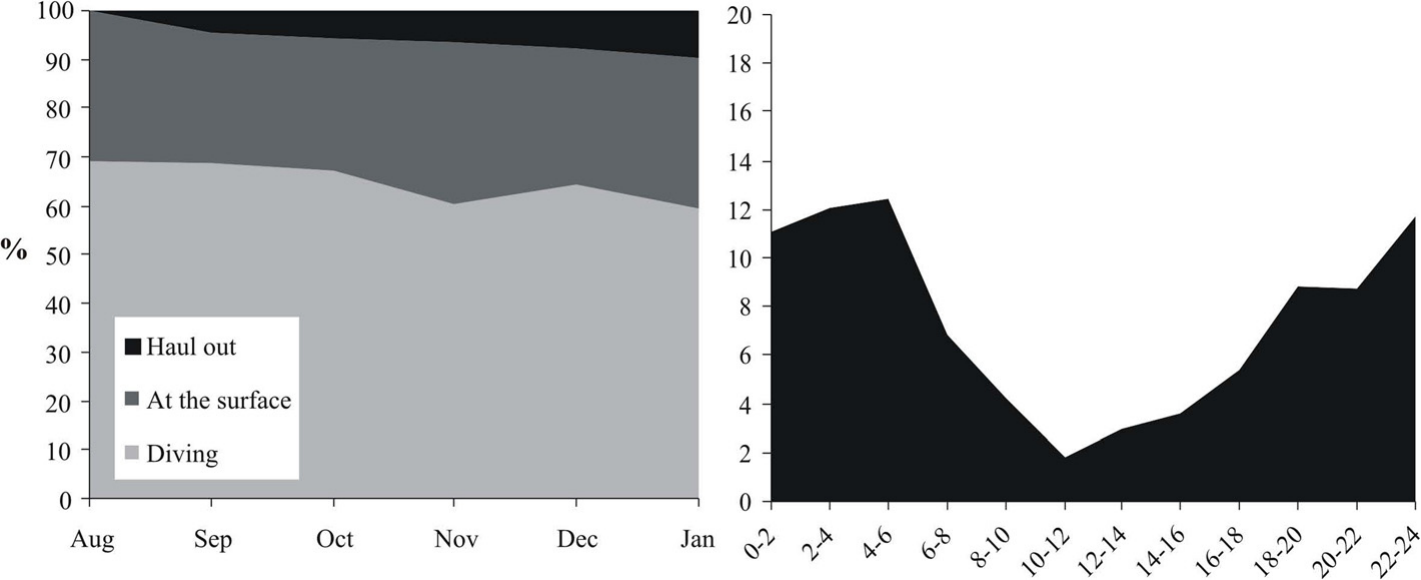
Time budget (left panel) and times of haul out (right panel) for Baltic ringed seals. Time frame: August-January, years 2011–2014. Tracked seals: 26 individuals. Time is local time (UTC + 2)

During the foraging season (Aug–Jan), 41 out of 79 tracks had a peak in the variance of log(FPT), indicating increased search effort at scales varying from 2.5 to 60.5 km (mean 13.5 ± 14.7 km). Foraging areas could not be identified for two individuals (ME11, PI12), which did not show increasing search effort at any scale and were, therefore, moving randomly. The other 24 seals had from 1 to 6 foraging areas (mean 3.1 ± 1.6, Fig. 3) and they spent 47 ± 22 % of time inside these zones. Typically foraging areas of individuals had a mean distance of 254 ± 194 km. However, the distance between foraging areas had large variation among individuals: 9 seals were relatively local foragers having only one foraging area or the mean distance between several foraging areas was 67 ± 26 (range 35–100) km. The other 17 seals were “long-range foragers”, which had either several separate foraging areas (mean distance 328 ± 180 km, range 150–825 km) or no main foraging areas could be detected. Each tracked ringed seal used 26 ± 16 haul out sites (range 0–55), 59 ± 30 % of which were inside the foraging areas. Haul out consisted 7.5 % of the time budget during the foraging season and was mainly nocturnal (Fig. 4).

**Fig. 5:**
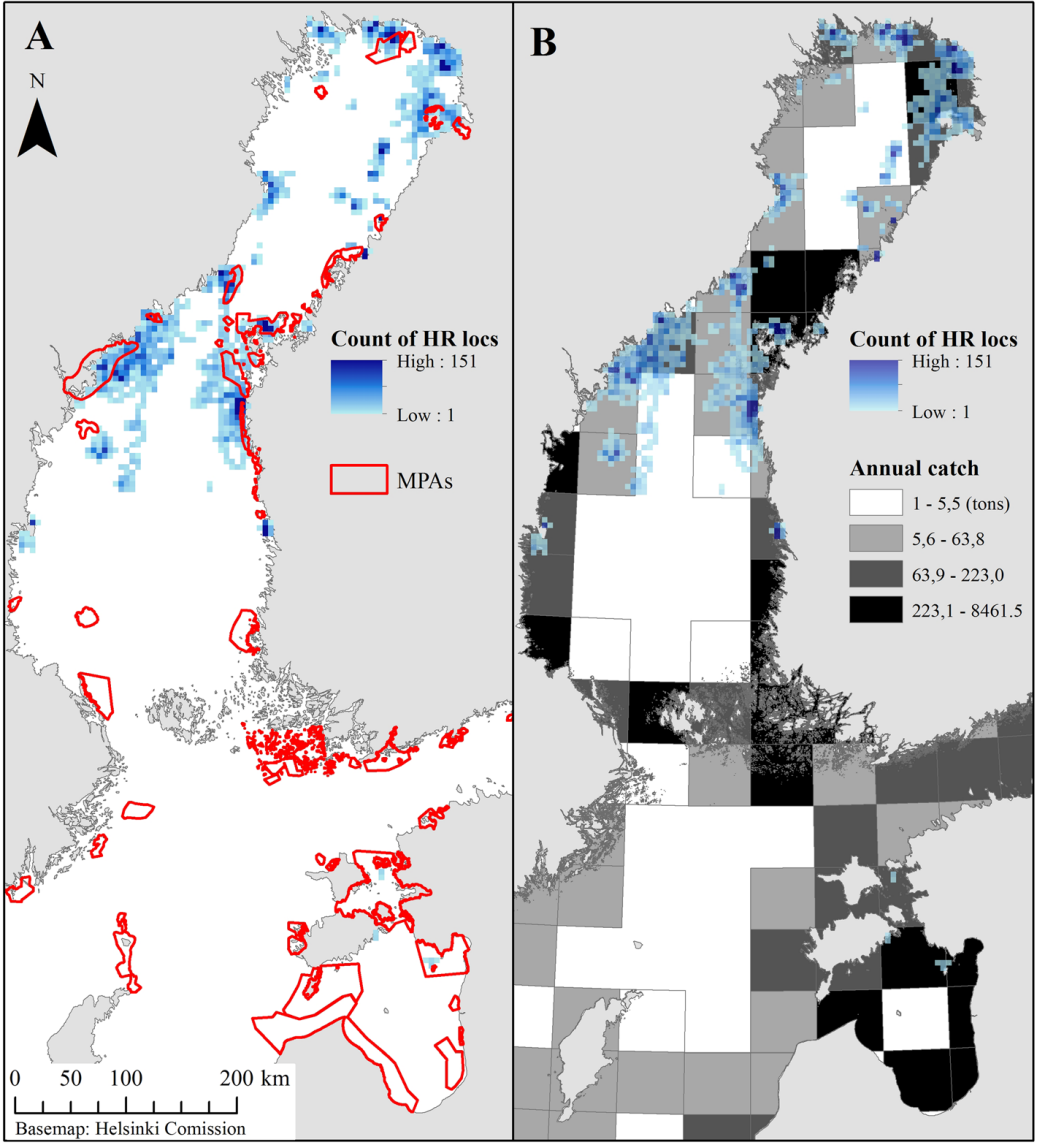
Overlap of high residency locations of Baltic ringed seals with marine protected areas (**a**) and coastal fisheries (**b**). Count of high residency (HR) locations in 5 × 5 km grids for tracked ringed seals (*n =* 26). Time frame: August-January, years 2011–2014. Annual catch of coastal fisheries is in tons of kg for year 2007

Despite the high number of long-range foragers among the tracked seals, two clusters of foraging “hot spots” were identified; one in the northern Bothnian Bay and another in the northern Bothnian Sea and the Quark (Figs. 3 and 5). The foraging areas were characterized by a shallow bathymetry (median depth of high residency locations 13 ± 49 m [mean 38 m]) and proximity to the shore (median distance from the mainland 10 ± 14 km [mean 15 km]). Overall, 22 % of high residency locations were situated within the existing protected areas (19 % to MPAs and 15 % to Natura 2000 sites) and 47 % overlapped with areas where annual catch of coastal fisheries were over the median value (63.8 tons of kg) (Fig. 5).

## Discussion

The present study is the first to document extensive movements of Baltic ringed seals. The tracked seals utilised on average 27 % (MCP home ranges 31 565 ± 16 640 km^2^) of the surface area of the Gulf of Bothnia (115 500 km^2^, [37]). The distances that Baltic ringed seals ranged from the tagging site (mean 392 km) were similar to Arctic ringed seals that range over distances of several hundreds of kilometres during the post-moulting season [16, 27, 28, 53–55]. However, Arctic ringed seals reportedly travel a couple of thousand kilometres from the tagging site [16, 26, 56]. The estimated home ranges of the present study (8030 km^2^, 95 % a-LoCoH) were similar to those reported for ringed seals in the eastern Canada (“locals” 2281 and “long rangers” 11 854 km^2^, [57]). In contrast, ringed seals in Lake Saimaa have very modest home ranges (92 km^2^, [30]), likely due to the complex structure of the small lake habitat (area 4400 km^2^, [58]). The home ranges reported here match the average home ranges of the Baltic grey seals (*Halichoerus grypus*, 6294 km^2^ [59] and 6858 km^2^ [10]), which are known to move long distances over the whole Baltic Sea. Although the home range sizes for Baltic ringed seals have not been previously reported, they have been considered quite sedentary due to the limited movements observed in the previous study [25]. However, our observations indicate that the movements of ringed seals in the Baltic Sea are similar order of magnitude to those in the Arctic Sea. In addition, also genetic results [28, 60] have indicated that Baltic ringed seals may be more mobile than earlier suggested.

The results of the present study suggest that during breeding season adults are mostly associated with good ice conditions whereas juveniles are near the ice edge or in the open-water areas. Baltic ringed seals may therefore exhibit similar habitat partitioning between adults and juveniles during the breeding season as reported in the Arctic [61]. Whereas the GPS phone tags of juveniles were mostly working well during breeding season, tags of adults ceased to work or only transmitted very few locations when they moved to ice-covered areas in January-February. However, the last obtained locations from the breeding season indicate that most adults occupied the ice-covered areas in the northern Bothnian Bay and the Gulf of Riga, which are the main breeding areas for the Baltic ringed seals and characterised by the presence of pack and stable ice during most winters [62]. Two adult females migrated from the Bothnian Bay to the Gulf of Riga in November-December, suggesting that some individuals move between different subpopulations. Ringed seals show breeding site fidelity [16, 19] and it is likely that these individuals were feeding in the Bothnian Bay and returned to breed to the Gulf of Riga. The frequency of the movements between breeding areas on the population level remains unclear.

Our results confirm the previous observations of nocturnal haul out behaviour during the post-moulting for the Baltic ringed seal [25]. The Saimaa seal also has similar nocturnal haul out rhythm [21, 29, 63]. In contrast, ringed seals in Greenland have not shown any circadian rhythm in their haul out behaviour [20, 53]. Tracked ringed seals hauled out only 8 % of their total time, which is quite similar to the 10 to 17 % previously reported for ringed seals during the post-moulting season [16, 25, 63]. The observed low proportion of time spent hauling out indicates that haul out contributes relatively little to the high residency areas (referred to as foraging areas) estimated with the FPT approach. Ringed seals can also sleep in the water [64], and at-sea activities may include some of this resting behaviour as well. However, as the open-water season is the most important foraging time when ringed seals gain considerable weight [23–25], the high residency areas very likely refer to the areas of increased foraging effort.

Baltic ringed seals used large regions for foraging. Most (65 %) of the tracked ringed seals were “long-range” foragers that used spatially remote foraging areas or did not concentrate foraging efforts to any particular area. Foraging near the mainland (median distance 10 km) in areas with shallow bathymetry (depth 13 m) indicates potential overlap and interactions with coastal fisheries. Ringed seals are suggested to cause substantial catch losses to the coastal fisheries in the Bothnian Bay, although grey seals induce most damage at the scale of the Baltic Sea [33, 34, 65]. Removal of ringed seals near the fishing gear in the Bothnian Bay has been proposed to mitigate the depredation [35]. As most of the ringed seal individuals seem to feed on relatively large areas within the foraging season, our results indicate that removal of the individuals near the fishing gear may not be locally effective method to mitigate the ringed seal-induced damages to coastal fishery. Furthermore, due to the extensive movement capacities, local mitigation actions may target individuals from the southern subpopulations and therefore compromise conservation goals in these areas, further complicating the management of the conflict.

Despite the extensive movements and large proportion of long range foragers, two clusters of ringed seal foraging “hot spots” were identified, one in the Quark and the other in the northern Bothnian Sea. According to old bounty statistics, ringed seals gather to the northern Bothnian Bay in the late fall [66], when we also captured mostly adults with the seal nets. Their foraging areas were more clearly clustered to the northern Bothnian Bay compared to juveniles. The juveniles were mainly captured in fyke nets earlier in fall, which is in line with the by-catch records [38]. The foraging areas of the tracked seals partly overlapped with MPAs and Natura 2000 sites especially in the identified foraging hot spots. Both protected area networks aim to conserve important species and habitats, ringed seal being one of those species [52, 67]. However, ringed seal was listed as criteria for protection in 7 out of 15 MPAs and in only 5 out of 30 Natura 2000 sites that overlapped with high ringed seal residency [67, 68]. Our results therefore indicate that safeguarding of the important resting and feeding habitats could to some extent be implemented in and adjacent to the existing protected area networks. Consequently, identified foraging areas of ringed seals should be taken into account when updating the management plans for overlapping protected areas. Importance of the Bothnian Bay as the main distribution and breeding area of the Baltic ringed seal may be emphasized in the future, as the warming climate reduces ice cover and thereby the breeding success of the southern subpopulations [15, 31]. Therefore, the future conservation measures may need to be directed more strongly towards the subpopulation of the Bothnian Bay. In general, marine mammals rely on healthy ecosystems for their survival and they are indicators of marine ecosystem change and biodiversity [69]. The foraging distribution of ringed seals might therefore be utilised also as indicators for identifying important areas for protection.

The chosen analytical approach, including position filtering, linear interpolation of the tracks and first passage time analyses, was heuristic rather than statistical [70]. However, our results and conclusions should be quite robust to the weaknesses of these approaches, given the accuracy of the GPS positions, large number of daily fixes (17 ± 8 locations/d) and the study questions related to the broad-scale habitat use. In the future, however, more fine-scaled analyses on foraging behaviour and habitat preference of the Baltic ringed seal, based on state-space methods, for example, are encouraged.

## Conclusions

The foraging of Baltic ringed seals is mostly concentrated to relatively shallow areas near the mainland, indicating potential overlap with coastal fisheries. The conflict between ringed seals and coastal fisheries has intensified in the Bothnian Bay as the seal population has been recovering. The mitigation of the conflict is complex, as ringed seals range over large areas and concentrate to forage to different—often remote—areas. Selective removal of seals near the fishing gear may not therefore be the most suitable method to mitigate the depredation. On the other hand, clusters of foraging effort hot spots were identified. The hot spots overlapped partly with the existing protected areas. The importance of Bothnian Bay as the main distribution area may further increase due to changing climate, and the management of key foraging and resting habitats of ringed seals could to some extent be established within the existing network of protected areas.

## Additional file

Additional file 1: Table S1. Details of the Baltic ringed seals equipped with GPS phone tags. * : Individuals tagged additionally with SPOT5 flipper tags. Values in the brackets describe the date of last location and number of locations obtained with flipper tags. (PDF 86 kb)

## Abbreviations

a-LoCoH: Adaptive local nearest neighbour convex cull
FPT: First passage time
HELCOM: Helsinki comission
MCP: Minimum convex polygon
MPA: Marine protected area designated by HELCOM
Natura 2000 site: Network of protected areas, protection is based on the EU’s habitats directive
var-max: The maximum variance of log-transformed first passage time values
