# Supplementary material for: Identifying foraging habitats of Baltic ringed seals using movement data
Source: Mov Ecol. 2015 Sep 23;3(1):33. doi: 10.1186/s40462-015-0058-1 (PMC4580415; doi:10.1186/s40462-015-0058-1)
Supplement: Additional file 1: Table S1. — Details of the Baltic ringed seals equipped with GPS phone tags. * : Individuals tagged additionally with SPOT5 flipper tags. Values in the brackets describe the date of last location and number of locations obtained with flipper tags. (PDF 86 kb) [file 40462_2015_58_MOESM1_ESM.pdf]

	ID	Capture method	Sex	Age class	Weight (kg)	Capturing date	Last GPS location	No. Locations	Duration (d)
1.	SI11	fyke net	♀	juv.	38	2.9.2011	25.1.2012	3124	145
2.	ME11	fyke net	♀	juv.	50	22.9.2011	27.3.2012	1533	187
3.	IN12	fyke net	♀	juv.	42	29.8.2012	4.2.2013	3354	159
4.	MI12	fyke net	♀	juv.	47	22.9.2012	1.4.2013	6453	191
5.	PI12	fyke net	♀	adult	66	6.10.2012	23.10.2012	639	17
6.	SA13	fyke net	♂	juv.	45	19.9.2013	9.4.2014	2093	202
7.	VA13	fyke net	♀	juv.	45	24.9.2013	19.2.2014	1873	148
8.	AA13	fyke net	♂	juv.	43	12.10.2013	22.1.2014	1968	102
9.	AS13	fyke net	♀	juv.	44	2.11.2013	24.3.2014	2328	142
10.	RE13	fyke net	♂	juv.	42	5.11.2013	27.4.2014	4008	173
11.	SU13	seal net	♀	juv.	42	4.11.2013	18.2.2014	1453	106
12.	KU13	seal net	♂	juv.	41	6.11.2013	3.4.2014	336	148
13.	HE11	seal net	♀	adult	103	7.11.2011	11.2.2012	1160	96
14.	TE11	seal net	♂	adult	100	9.11.2011	23.1.2012	782	75
15.	EL11	seal net	♀	adult	82	9.11.2011	28.11.2011	516	19
16.	VI11	seal net	♀	juv.	40	13.11.2011	4.5.2012	1762	173
17.	II11	seal net	♀	adult	113	13.11.2011	7.2.2012	791	86
18.	LE11	seal net	♀	adult	108	14.11.2011	16.3.2012	2030	123
19.	PA12*	seal net	♀	adult	80	6.11.2012	7.2.2013 (5.5.2013)	1891 (97)	93
20.	EI12*	seal net	♂	adult	91	18.11.2012	9.3.2013 (5.5.2013)	1951 (42)	111
21.	EI13*	seal net	♀	adult	102	31.10.2013	28.2.2014 (9.11.2013)	3152 (1)	120
22.	MA13	seal net	♂	adult	96	9.11.2013	9.2.2014	2218	92
23.	TE13*	seal net	♂	adult	120	9.11.2013	11.2.2014 (2.5.2014)	882 (21)	94
24.	KA13	seal net	♀	adult	64	10.11.2013	5.2.2014	832	87
25.	IL13	seal net	♂	adult	91	14.11.2013	27.1.2014	926	74
26.	JA13	seal net	♀	adult	60	16.11.2013	11.3.2014	1075	115
